# Integrated machine learning and multi-dataset analysis identify SDS as a diagnostic and immune-related hub gene in hepatoblastoma

**DOI:** 10.3389/fonc.2026.1895612

**Published:** 2026-09-30

**Authors:** Dan Chen, Baixue Zhang, Yanlong Zhang, Na Li, Ang Li, Wei Gao

**Affiliations:** 1 Department of Pediatric Hematology and Oncology, Xingtai People’s Hospital, Xingtai, China; 2 Department of Oncology, Second Affiliated Hospital of Xingtai Medical College, Xingtai, China; 3 Department of Cardiology, Xingtai People’s Hospital, Xingtai, China; 4 Department of Public Health, Jining Medical University, Jining, China; 5 School of Public Health, North China University of Science and Technology, Tangshan, China

**Keywords:** diagnostic markers, hepatoblastoma, immune microenvironment, machine learning, SDS

## Abstract

**Background:**

Current clinical diagnostic models lack sufficient sensitivity and specificity for Hepatoblastoma (HB) individualized prediction. This study aimed to identify robust hub genes for HB diagnosis and molecular subtyping by integrating multiple transcriptomic datasets and machine learning algorithms.

**Methods:**

Four merged GEO hepatoblastoma datasets (GSE131329, GSE132037, GSE104766, GSE133039) were analyzed by differential expression and WGCNA to identify HB-related genes. Further use three algorithms (LASSO, SVM-RFE, Random Forest) to identify central genes. In addition, consensus clustering was used for molecular typing, and four deconvolution algorithms (TIMER, MCPcounter, xCell, quanTIseq) were used to characterize the tumor microenvironment. The biological function of SDS in hepatoblastoma was elucidated through *in vitro* experiments (CCK-8 detection, colony formation assay, Transwell assay), as well as *in vivo* using a xenograft mouse model.

**Results:**

A total of 268 differentially expressed genes (77 up, 191 down) were identified. WGCNA revealed the blue module as most correlated with HB (correlation coefficient 0.66). Three machine learning algorithms jointly selected four hub genes (CLEC1B, ITGA6, SDS, TTC36). Consensus clustering based on these four genes stratified HB into two subtypes (Clust1, Clust2). Clust1 exhibited enhanced immune infiltration and activation of complement, IFN-α, and IL6-JAK-STAT3 pathways. Low SDS expression associated with cell cycle pathways, whereas high SDS expression associated with immune-related pathways. Functionally, SDS overexpression significantly suppressed HB cell proliferation, migration, invasion, and colony formation, while promoting apoptosis *in vitro*, and markedly reduced tumor growth *in vivo*.

**Conclusion:**

Integrated machine learning identified CLEC1B, ITGA6, SDS, and TTC36 as robust diagnostic genes for hepatoblastoma. SDS, in particular, exerts a tumor-suppressive role and is closely linked to the immune microenvironment, making it a promising diagnostic biomarker and therapeutic target in HB.

## 1 Introduction

Hepatoblastoma (HB) is the most common primary liver cancer in the pediatric population (1), responsible for about 80% of malignant liver tumors in children, with the peak incidence occurring in children under three years of age. Globally, the incidence rate of hepatoblastoma is increasing more rapidly than that of any other childhood cancer (2). Although patient outcomes have improved, with an overall survival rate exceeding 70%, individual responses to conventional treatment vary considerably, and the prognosis for high-risk HB remains significantly poor (3–5).

Currently, the diagnosis of HB largely depends on clinical and pathological indicators such as tumor stage, serum alpha-fetoprotein (AFP) levels, and imaging results. Some studies have also proposed that inflammatory markers, such as the neutrophil-to-lymphocyte ratio and platelet-to-lymphocyte ratio (PLR), can help refine prognostic assessments in clinical settings (6, 7). To improve diagnostic accuracy, many researchers have turned to large-scale databases, including the Surveillance, Epidemiology, and End Results (SEER) program, the Children’s Hepatic Tumors International Collaboration (CHIC), and other population-based cohorts, to develop clinical feature-based nomograms for HB (8–11). However, despite these efforts, models built solely on clinical features often fail to deliver the sensitivity and specificity needed for truly individualized predictions. This limitation undermines their reliability in forecasting patient outcomes and guiding personalized therapy. As a result, there is a clear and urgent need to better understand the key biological mechanisms driving HB. Such insights could lead to the discovery of reliable, sensitive biomarkers for early detection and open up new therapeutic avenues for patients with HB.

A number of genetic alterations have been tied to hepatoblastoma in previous studies, including changes in the expression levels of DLK1, MEG3, TET1, HMGA2, DKK1, DKK4, APC, AXIN1, AXIN2, and LGR6 (12–15). Additionally, hepatoblastoma is genetically characterized by aberrant Wnt signaling activation (16). Several genomic profiling studies of hepatoblastoma have demonstrated a high prevalence of Wnt-activating mutations in the majority of HB cases (17, 18). However, hepatoblastoma exhibits one of the lowest mutational burdens among all cancers (19), which has hindered the discovery of potential therapeutic targets for this disease. Machine learning algorithms have recently proven useful for analyzing large-scale datasets encompassing clinical, laboratory, and biomarker information.

Notably, novel models integrating multiple machine learning techniques have demonstrated significantly improved predictive performance compared to models using a single machine learning approach or traditional models based solely on clinical data (20).

Based on the above background, we integrated different hepatoblastoma datasets and further combined three machine learning algorithms-random forest (RF), LASSO regression, and support vector machine-recursive feature elimination (SVM-RFE)-to screen for key hub genes. Our aims to offer both a theoretical and experimental basis for molecular subtyping and targeted therapy in hepatoblastoma.

## 2 Materials and methods

### 2.1 Data acquisition and preprocessing

In this study, four Gene Expression Omnibus (GEO) datasets (GSE131329, GSE132037, GSE104766, and GSE133039) were selected as the training set, while GSE75271 served as the external validation set. mRNA expression matrices were obtained from these datasets. Probes that hybridized to more than one transcript were discarded, keeping only those with the strongest signal for each gene. All microarray data are first subjected to background correction and missing value imputation using RMA; Then, the differences between different samples are calibrated by quantile normalization to ensure comparability of the expression profiles of different samples. Finally, ComBat (sva) was used to perform batch effect correction on the integration of four GEO datasets, resulting in the final 240 samples (94 controls, 146 cases). **Table 1** provides an overview of the baseline characteristics for each dataset.

**Table 1:** Basic information of GEO hepatoblastoma datasets.

GEO datasets	Platform	Disease	Sample		Years	Country
			Control	Case		
GSE131329	GPL6244	Hepatoblastoma	14	53	2022	Japan
GSE132037	GPL17586	Hepatoblastoma	18	32	2020	Spain
GSE104766	GPL16791	Hepatoblastoma	30	30	2019	France
GSE133039	GPL16791	Hepatoblastoma	32	31	2020	Spain
GSE75271	GPL570	Hepatoblastoma	5	50	2019	USA

### 2.2 Data processing and differential gene analysis

We screened for differentially expressed genes (DEGs) between hepatoblastoma and control groups with limma, applying an adjusted *P* < 0.05 and |log2(fold change)| ≥ 2 as cutoffs. Up regulated and downregulated key genes were displayed in a volcano plot.

### 2.3 Weighted gene co-expression network analysis

WGCNA was applied to find gene modules in hepatoblastoma samples (cut height = 0.25, minimum module size = 50, soft power = 20, scale-free R² = 0.9). We calculated Gene significance (GS) and module membership (MM) and used Pearson rank correlation to assess module–trait relationships. This analysis pinpointed key hepatoblastoma-associated features and allowed visualization of the eigengene network.

### 2.4 Pathway and ontology enrichment

GO functional annotation and KEGG pathway enrichment analyses were performed using the clusterProfiler R package (version 4.0), the GO analysis covered biological process (BP), molecular function (MF), and cellular component (CC). To control the false discovery rate (FDR) in multiple hypothesis testing, the Benjamini-Hochberg (BH) method was applied for p-value adjustment. Adjusted *P* values < 0.05 were considered statistically significant. Additionally, only terms with a minimum enrichment gene count (count ≥ 2) were retained for further interpretation.

### 2.5 Machine learning algorithms

Feature genes were identified using LASSO, SVM-RFE, and random forest. LASSO regression via glmne selected genes at the optimal lambda.1se determined by ten-fold cross-validation. SVM-RFE via e1071 picked the best classification variables, while random forest via randomForest evaluated feature importance by averaging across multiple decision trees, offering robustness to noise. The genes common to three algorithms were retained as candidate diagnostic hub genes for hepatoblastoma.

### 2.6 Recognition of distinct subtypes by unsupervised clustering

Consensus clustering is an unsupervised machine learning approach that enhances the stability and reliability of clustering results. In this study, the R package “ConsensusClusterPlus” was used to identify subtypes of hepatoblastoma patients. The number of clusters was selected by evaluating the cumulative distribution function (CDF) curves, consensus matrices, and changes in the area under the CDF curve.

### 2.7 TME analysis

Four algorithms-Xcell, MCPcounter, quanTIseq, and TIMER-were used to infer immune cell composition in the TME from RNA-seq data. Differences in immune infiltration across groups were assessed and displayed in heatmaps. Subsequently, correlations between hepatoblastoma signature gene expression and both immune/stromal scores and immune cell subsets were further evaluated.

### 2.8 Gene set variation analysis and gene set enrichment analysis

We ran GSVA to evaluate pathway activity differences among groups and plotted significant pathways with ComplexHeatmap. To explore the biological significance of key genes, gene set enrichment analysis (GSEA) was then conducted via clusterProfiler. Reference gene sets (Hallmark v7.5 and KEGG v7.2) were sourced from MSigDB.

### 2.9 Real-time fluorescence quantitative PCR

Cells were lysed in Trizol for RNA extraction, and reverse transcription was performed with the RevertAid First Strand cDNA Synthesis Kit (Thermo Fisher Scientific). RT-qPCR was then carried out using SYBR Green Mix (Mei5 Biotechnology, Beijing, CHN). The 2^-ΔΔCt^ method was applied for relative quantification, using GAPDH for normalization.

### 2.10 CCK-8 assay

CCK-8 assays (Dojindo) were performed to measure cell viability. HuH6 and HepG2 cells (5 × 10³/well) were seeded in 96-well plates, cultured for the indicated times, treated with 10 µL CCK-8 for 1 h, and then analyzed at 450 nm.

### 2.11 Clone formation

For the colony formation assay, 1,000 cells from each cell line were evenly seeded into six-well plates and cultured at 37 °C in a 5% CO_2_ atmosphere. The culture medium was replaced every three days. After 14 days, the colonies were stained with a methanol solution containing 0.1% crystal violet for 30 minutes, then photographed and counted.

### 2.12 Cell migration and invasion assay

Migration and invasion were assessed by Transwell assays. Tumor cells were loaded into the upper compartment of Transwell inserts, with or without Matrigel coating. The lower compartment held RPMI 1640 supplemented with 10% FBS. Following a 24-h incubation, migrated and invaded cells were stained with 0.2% crystal violet for 20 min in the dark and visualized with an Olympus microscope.

### 2.13 Hoechst staining

Hoechst 33342 staining was performed to observe nuclear morphology of apoptotic cells. HuH6 and HepG2 cells transfected with empty vector or SDS overexpression plasmid were plated in 6-well plates (3 × 10^5^ cells/well) and incubated overnight at 37 °C in 5% CO_2_. After 48 h, we washed the cells with PBS, fixed them with 4% paraformaldehyde for 15 min at room temperature, and then stained with Hoechst 33342 (3 µg/mL, Solarbio) for 10 min in the dark. Subsequently, the stained cells were washed with PBS and examined under an IX71 fluorescence Olympus microscope at ×400 magnification.

### 2.14 Western blot analysis

Cells were lysed in RIPA buffer (Thermo Fisher Scientific, MA, USA), and protein concentrations were determined using a BCA kit(Solarbio, Beijing, China). Proteins were separated by 10% SDS-PAGE, transferred to nitrocellulose membranes, blocked with 5% skim milk, and incubated with primary antibody overnight at 4 °C. After TBST washes, membranes were incubated with HRP-conjugated secondary antibody, washed three times, and visualized using enhanced chemiluminescence. Primary antibody against SDS (ab68536) was from Abcam; β-actin served as the loading control.

### 2.15 Xenograft models

Male BALB/c nude mice at four weeks of age were randomly assigned to two groups, with five mice in each group. Huh6 cells (3 × 10^6^/0.2ml PBS) stably transfected with SDS and empty vector were subcutaneously injected into nude mice to establish xenograft model. Xenografts were examined every 3 days with digital calipers. After 23 days, the mice were sacri ficed and stripped the tumors for further analysis. All mice were bought from SPF Biotechnology (Beijing, China) and used in accordance with the agreement approved by the Animal Ethics and Welfare Committee of North China University of Science and Technology (2025-SY-3048-01).

### 2.16 Statistical analysis

Data were analyzed in R 4.2.3 and GraphPad Prism 10 (La Jolla, CA, USA). The Wilcoxon rank-sum test or Student’s t-test was applied for group comparisons, and values are given as mean ± SD. Pearson correlation assessed variable associations. A *P*-value below 0.05 was considered significant.

## 3 Results

### 3.1 DEGs and functional analysis

To discover differentially expressed transcripts associated with HB, we integrated bulk transcriptome sequencing samples from four datasets (GSE131329, GSE132037, GSE104766, and GSE133039) to construct a combined GEO-HB cohort. Batch effect correction and differential expression analysis were first performed on the merged data. To eliminate the prominent expression heterogeneity among samples, we used the ComBat algorithm to perform batch effect correction on the merged expression matrix of the dataset (**Supplementary Figure 1A**). In addition, we plotted UMPA plots before and after calibration. The samples before calibration were clearly clustered according to the source of the dataset, while after calibration, the samples in each dataset were evenly mixed. Further verifying the effectiveness of the calibration strategy at the data distribution level (**Supplementary Figure 1B**). Based on this, a total of 268 DEGs were identified, of which 77 transcripts were upregulated and 191 downregulated (**Figure 1A**).

**Figure 1:**
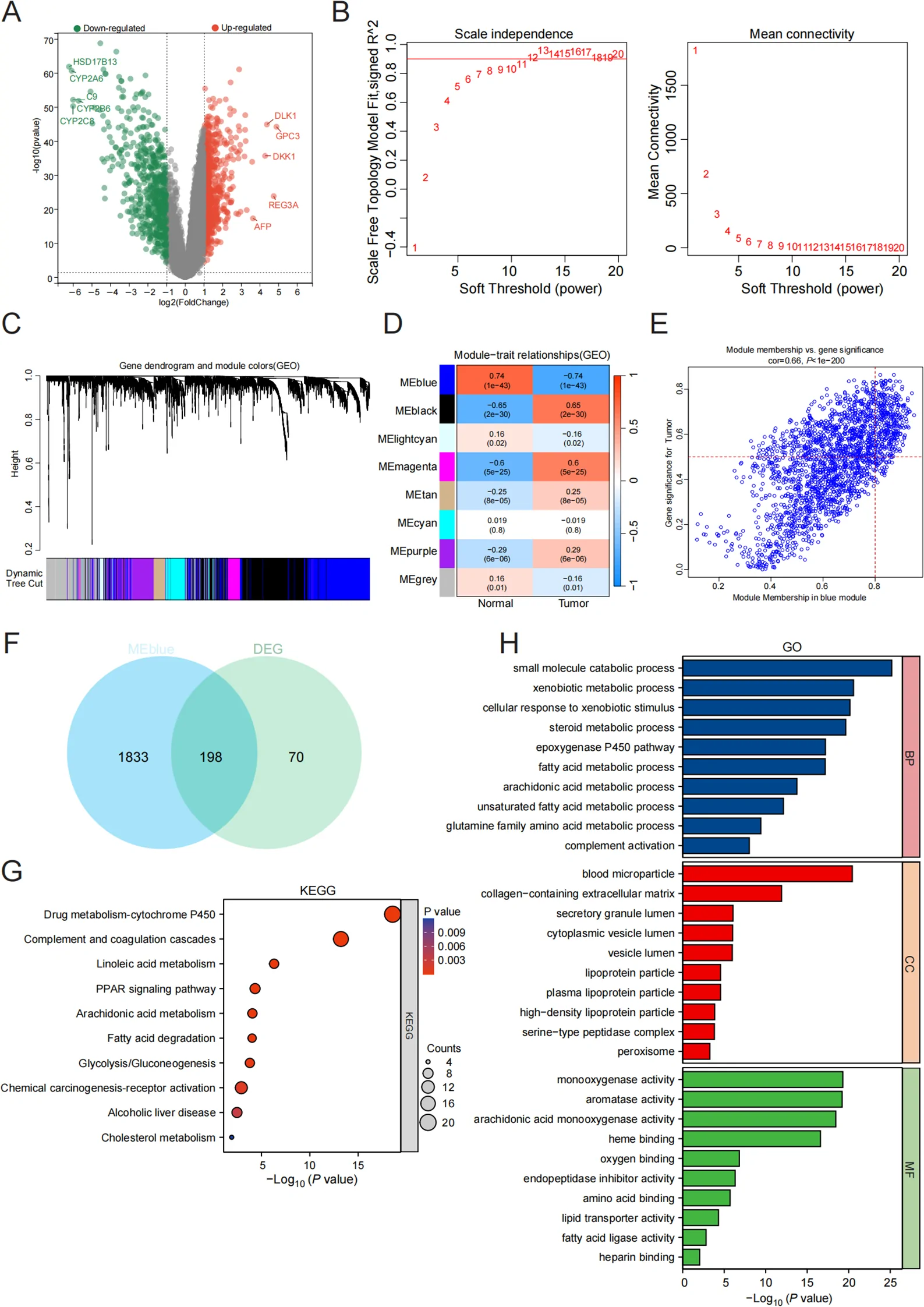
Screening of diagnostic genes for hepatoblastoma. **(A)** Volcano plot showing DEGs between HB and normal tissues. **(B)** Evaluation of different soft-thresholding powers based on mean connectivity and the scale-free fit index. **(C)** Clustering dendrogram of all genes generated using a dissimilarity algorithm and module assignment. **(D)** Heatmap visualizing correlations between modules and clinical traits. Red and green indicate positive and negative correlations, respectively. **(E)** Scatter plots for the blue module, illustrating the relationship between module membership and gene significance in HB. **(F)** Venn diagram of DEGs overlapping with blue module genes. **(G, H)** Enrichment analysis of the overlapping genes using GO and (KEGG pathway.

Subsequently, WGCNA was used to identify key modules closely associated with HB. A soft-thresholding power of 8 was selected to achieve a scale-free topology (**Figure 1B**), and correlations between module eigengenes and disease status were evaluated (**Figures 1C, D**). Eight co-expression modules were obtained, among which the blue module showed the strongest correlation with HB (correlation coefficient = 0.66, *P* < 1.0e^-200^; **Figure 1E**). The blue module was therefore taken forward as the focus of further investigation.

To further pinpoint core differential genes in HB, the 268 DEGs were intersected with the 2031 genes contained in the blue module, yielding 198 key HB-related genes (**Figure 1F**). To characterize the functional features of these candidate genes, KEGG pathway enrichment analysis and GO functional annotation were performed. The results showed that the most significantly enriched KEGG pathway was Drug metabolism-cytochrome P450 (**Figure 1G**), while GO enrichment analysis indicated that these genes are mainly involved in regulating the fatty acid metabolic process (**Figure 1H**).

### 3.2 Machine learning-based screening and performance evaluation of core diagnostic genes

To evaluate the diagnostic performance of each candidate gene in distinguishing HB samples, we ranked them based on the area under the receiver operating characteristic (ROC) curve. As shown in **Figure 2A**, the AUC values of the candidate genes were mainly distributed between 0.75 and 1.00. Using an AUC threshold greater than 0.95, we extracted 34 genes with relatively high diagnostic accuracy as a core candidate set. Subsequently, three machine learning algorithms RF, SVM-RFE, and LASSO were employed to identify genes closely associated with hepatoblastoma from these 34 genes. The RF analysis showed an overall prediction error of only 2.92%, after ranking by gene importance scores, the top 20 genes were selected (**Figures 2B, C**). LASSO regression with ten-fold cross validation revealed that the minimum binomial deviation corresponded to a penalty parameter -log(λ) of -2.756, at which nine genes had non-zero coefficients (**Figures 2D, E**). Meanwhile, SVM-RFE cross validation achieved a maximum accuracy of 0.97 with 29 feature genes, corresponding to an error of 0.03 (**Figures 2F, G**). Finally, the four hub genes commonly identified by all three machine learning methods were selected as core genes associated with hepatoblastoma: CLEC1B, ITGA6, SDS, and TTC36 (**Figure 2H**). The diagnostic value of these genes was further assessed using ROC curves. The AUC values for CLEC1B, ITGA6, SDS, and TTC36 were 0.952, 0.977, 0.971, and 0.965, respectively (**Figure 3I**). To further elucidate the advantages of our identified biomarkers over existing clinical indicators, we compared their area under the curve (AUC) values with that of AFP (AUC = 0.82), a clinical diagnostic biomarker for mature hepatoblastoma (**Supplementary Figure 2A**). The four core genes all yielded higher AUC values than AFP, indicating favorable predictive performance. In addition, the diagnostic value of the four core genes was evaluated using the external validation set GSE75271, and the results showed that these four genes maintained strong discriminatory performance in the external validation set (AUC>0.95), further supporting their reliability as potential biomarkers (**Supplementary Figures 2B–E**).

**Figure 2:**
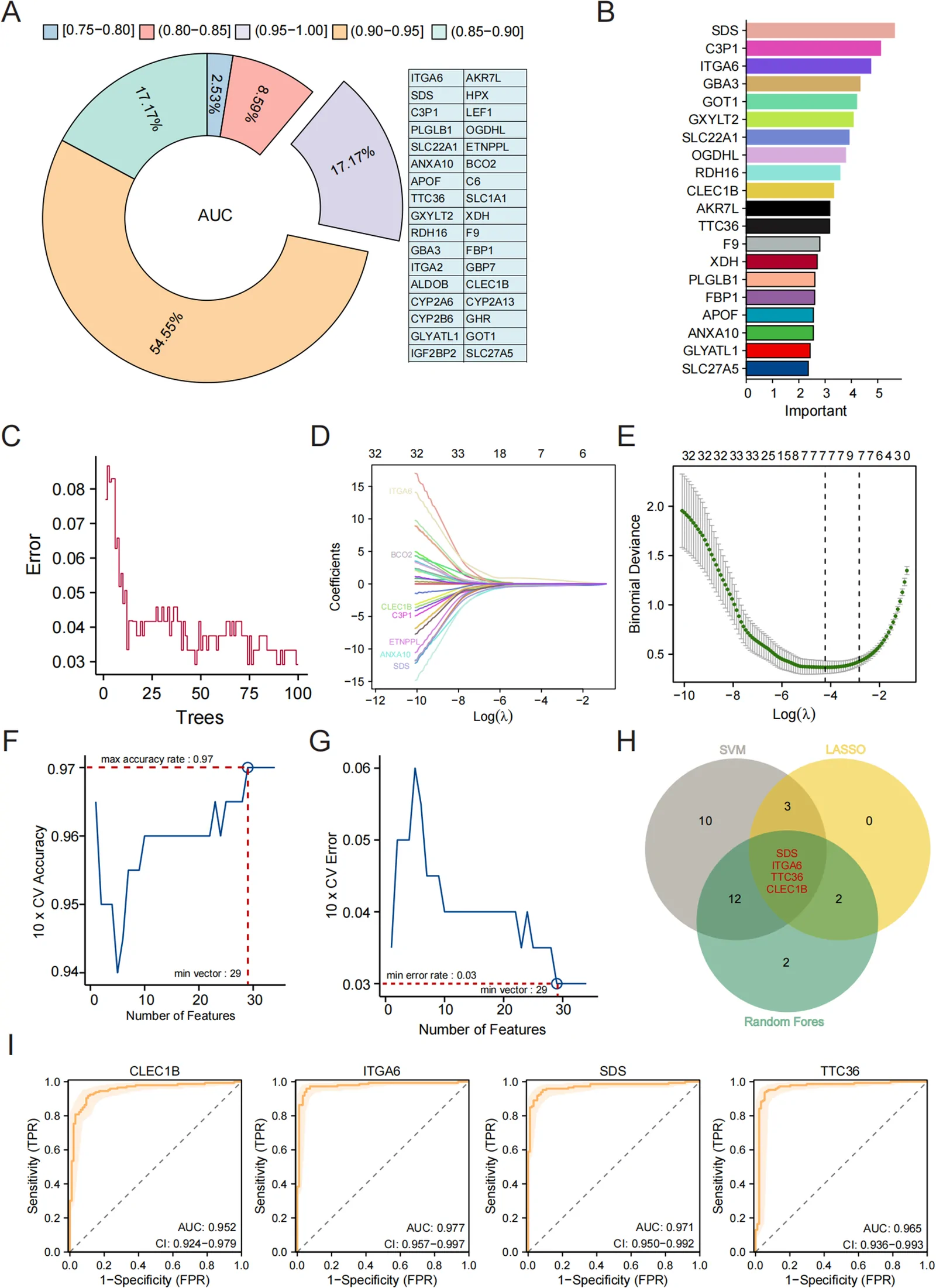
Multiple machine learning methods were used to further screen candidate genes. **(A)** Diagnostic performance evaluation of 198 candidate genes. **(B)** Diagram of hub gene importance in the RF algorithm. **(C)** Error-tree diagram of the RF algorithm. **(D)** LASSO coefficient profiles. **(E)** Relationship between -log(λ) and binomial deviance in the LASSO algorithm. **(F, G)** Accuracy and error of hub genes in the SVM-RFE algorithm. **(H)** Core genes identified by the intersection of LASSO, SVM-RFE, and RF algorithms. **(I)** ROC curves for evaluating the diagnostic performance of the feature genes.

**Figure 3:**
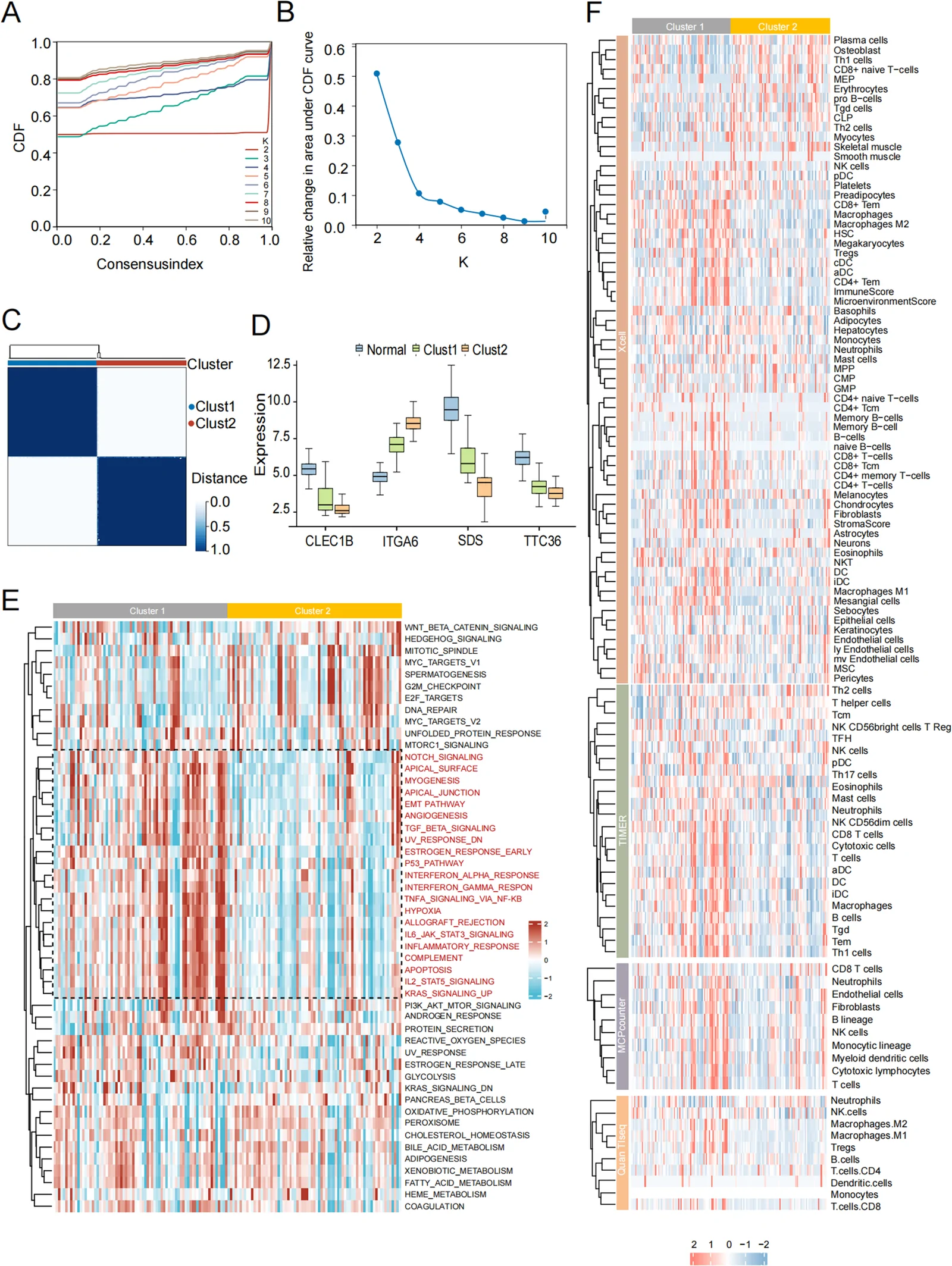
Hepatoblastoma subtype identification and pathway enrichment. **(A)** Cluster consensus matrix at k = 2. **(B)** CDF curves from k = 2 to 10. **(C)** Relative variation in delta CDF area. **(D)** Expression levels of CLEC1B, ITGA6, SDS, and TTC36 in normal tissues and different subtypes. **(E)** Pathway enrichment analysis of 50 hallmark gene sets between the two subtypes. **(F)** Immune cell infiltration enrichment in the two subtypes.

### 3.3 Molecular subtyping and immune landscape analysis based on key genes

Based on the four key genes identified above, consensus clustering was performed to classify 146 hepatoblastoma tissue samples into molecular subtypes. When the number of clusters was set to k = 2, a clear turning point was observed in the relative change in the area under the CDF curve, indicating that dividing the samples into two molecular subtypes was the optimal strategy (**Figures 3A–C**). Further analysis revealed that, compared with normal tissues, CLEC1B, SDS, and TTC36 were downregulated in tumor tissues, with even lower expression levels in the Clust2 subtype, whereas ITGA6 showed the opposite expression pattern (**Figure 3D**). Subsequently, using gene sets from the MSigDB database, GSVA was performed to calculate pathway activity scores for each sample, thereby evaluating the biological functions and pathway enrichment profiles. The results showed that the Clust1 subtype was enriched for more processes related to immune activation (complement, interferon-α response, IL-6/JAK/STAT3 signaling), apoptosis, and epithelial-mesenchymal transition (EMT) (**Figure 3E**). A joint analysis of immune infiltration using TIMER, MCPcounter, xCell, and QUANTISEQ revealed that Clust1 contained a higher level of infiltrating immune cells (**Figure 3F**).

### 3.4 Correlation analysis of SDS expression with the immune microenvironment

To preliminarily explore the potential associations between the expression levels of CLEC1B, ITGA6, SDS, and TTC36 and the immune infiltration characteristics of hepatoblastoma, correlation analyses were performed. The results demonstrated that, compared with the other three genes, SDS expression exhibited the strongest correlations with immune cell abundance (**Figures 4A–D**). Concurrently, SDS expression showed relatively high correlations with ESTIMATE score, StromalScore, and ImmuneScore (*r* = 0.517, 0.473, and 0.509, respectively; all *P* < 0.001; **Figures 4E–H**). These findings suggest that SDS expression status may be closely associated with the composition of the tumor microenvironment in hepatoblastoma, implicating it as a potential candidate gene involved in microenvironmental regulation.

**Figure 4:**
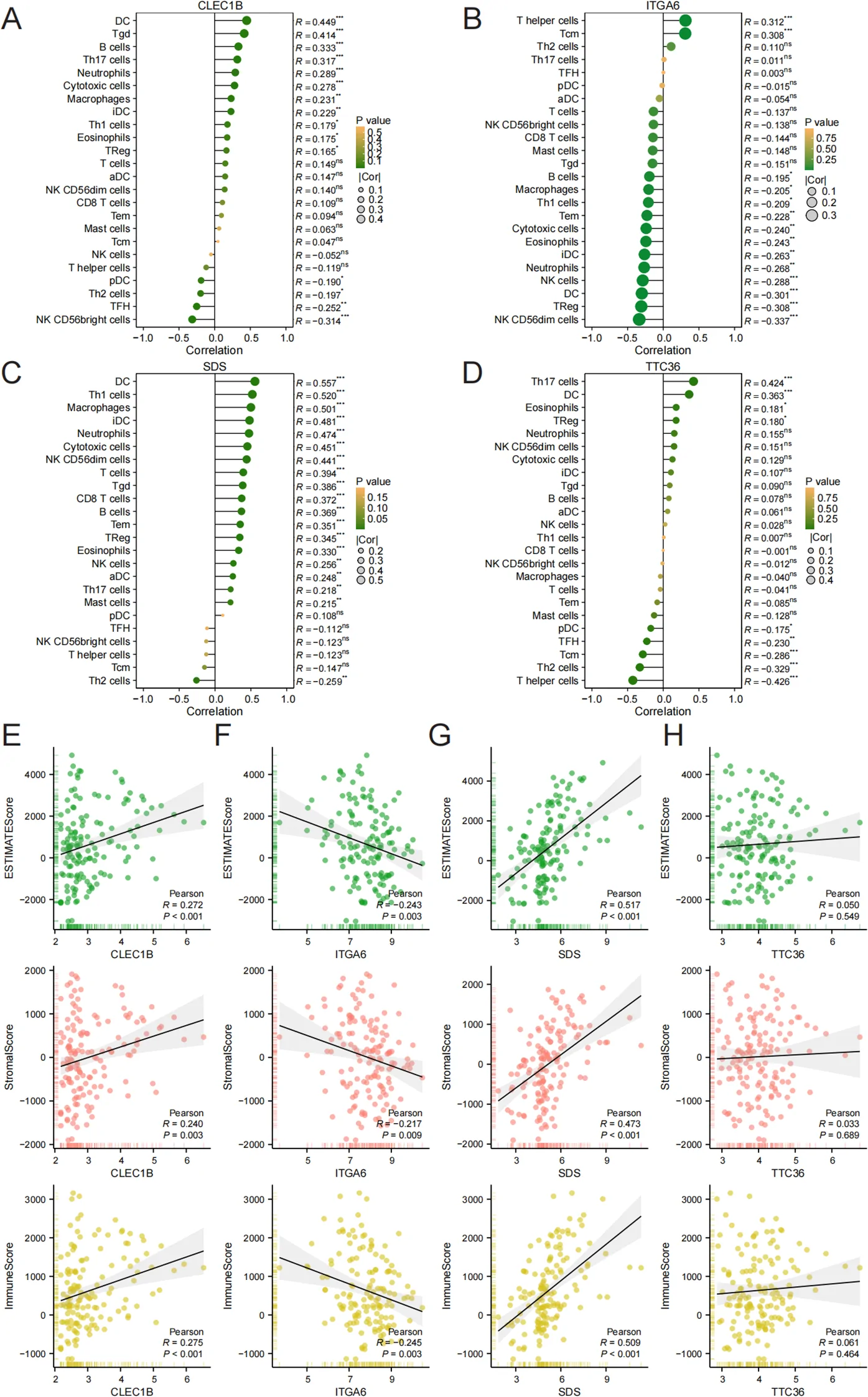
Prediction of immune processes based on expression of four candidate genes. **(A–D)** Correlation analysis between the expression of CLEC1B, ITGA6, SDS, and TTC36 and immune cell infiltration in hepatoblastoma. **(E–H)** Expression of CLEC1B, ITGA6, SDS, and TTC36 versus StromalScore, ImmuneScore, and ESTIMATE Score, respectively. Data are the mean ± SD. ^*^P < 0.05, ^**^P < 0.01, ^***^P < 0.001; ns, not significant.

To further investigate the differences in molecular profiles between groups with high and low SDS expression, we conducted GSVA enrichment analyses using the KEGG and Hallmark gene sets. The results indicated that the low-SDS expression group was mainly enriched in cell cycle and proliferation-related pathways (e.g., G2/M checkpoint, mitotic spindle, E2F targets), whereas the high-SDS expression group displayed prominent enrichment in several immune-related pathways (including IL6-JAK-STAT3 signaling, interferon response, and complement system) (**Figures 5A, B**). Based on these enrichment patterns, we postulate that low SDS expression may be associated with enhanced proliferative activity of tumor cells, whereas high SDS expression tends to coincide with the activation of immune-related pathways. Collectively, our bioinformatics analyses reveal a robust statistical association between SDS and the immune microenvironment of hepatoblastoma, providing clues for its potential involvement in immune regulation. However, these findings warrant further validation through functional experiments.

**Figure 5:**
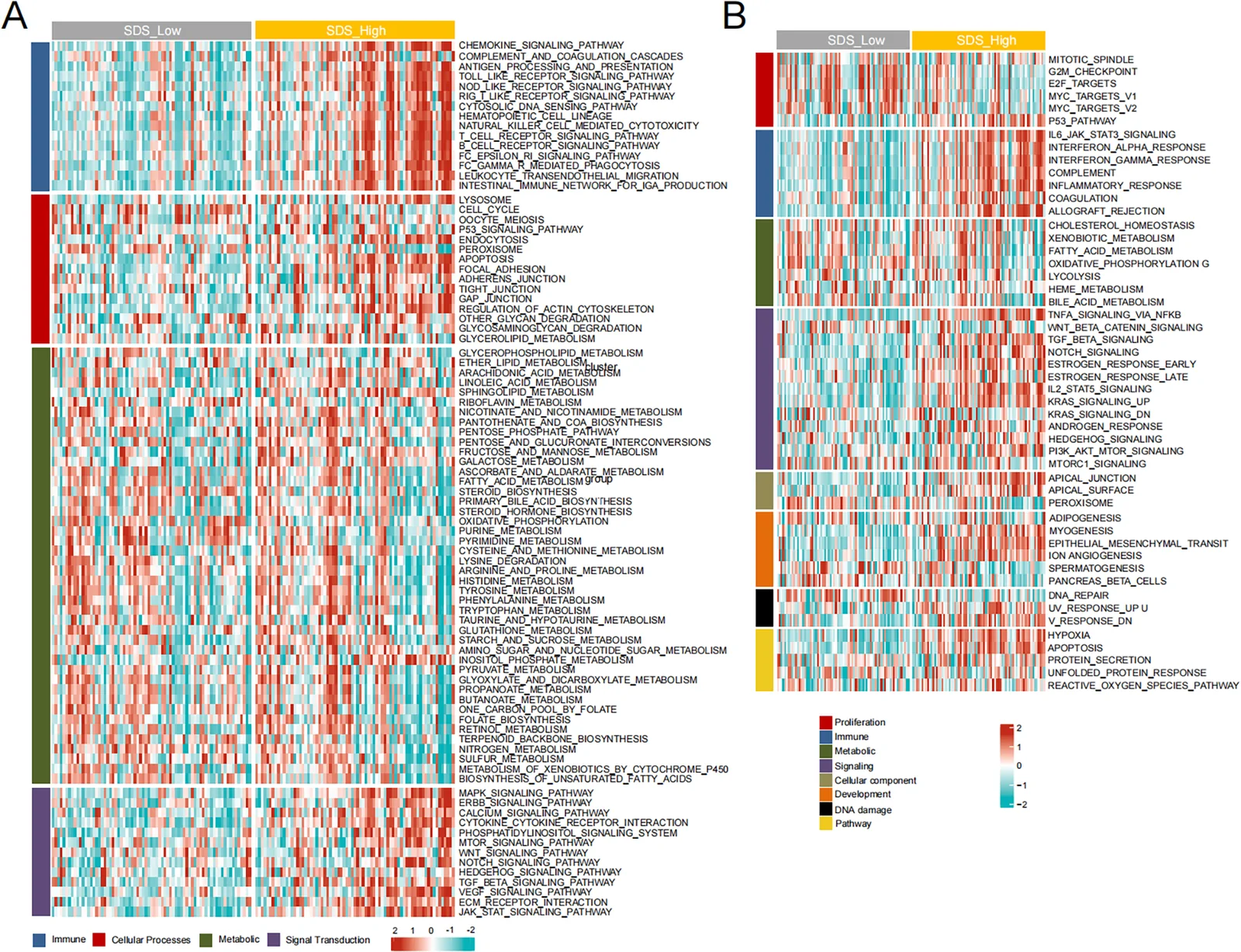
Pathway enrichment analysis based on SDS high- and low-expression groups. **(A)** Functional enrichment across 50 Hallmark gene sets. **(B)** Pathway enrichmentusing KEGG gene sets.

### 3.5 Effect of SDS overexpression on the biological functions of hepatoblastoma cells

To investigate the biological function of SDS in hepatoblastoma, hepatoblastoma cells were transfected with an SDS overexpression plasmid. At 24 hours post-transfection, the mRNA and protein expression levels of SDS significantly increased (**Figures 6A, B**). Functional assays revealed that SDS overexpression markedly inhibited cell proliferation, as confirmed by both colony formation and CCK-8 assays (**Figures 6C, D**). At the mechanistic level, GSEA based on HALLMARK and KEGG gene sets indicated that SDS expression was positively correlated with gene sets promoting apoptosis (**Supplementary Figure 3A**). Hoechst staining analysis further notable changes in hepatoblastoma cells nuclear morphology were observed (**Figure 6E**). In addition, GSEA also suggested that high SDS expression is associated with activation of EMT and cell adhesion pathways (**Supplementary Figure 3B**). Transwell assays and wound healing showed that SDS overexpression suppressed tumor cell migration and invasion (**Figures 6F, G**). Conversely, we silenced SDS expression using siRNA in HB cells. CCK-8 and Transwell assays revealed that silencing SDS significantly promoted HB cell proliferation, migration and invasion(**Supplementary Figures 4A, B**). To investigate the roles of SDS in HB tumorigenesis *in vivo*, Huh6 cells with upregulated SDS were subcutaneously injected into BALB/c mice. We found that the mean volume and weight of tumors in the SDS overexpression groups were substantially lower than those from the control group (**Figures 6H–J**).

**Figure 6:**
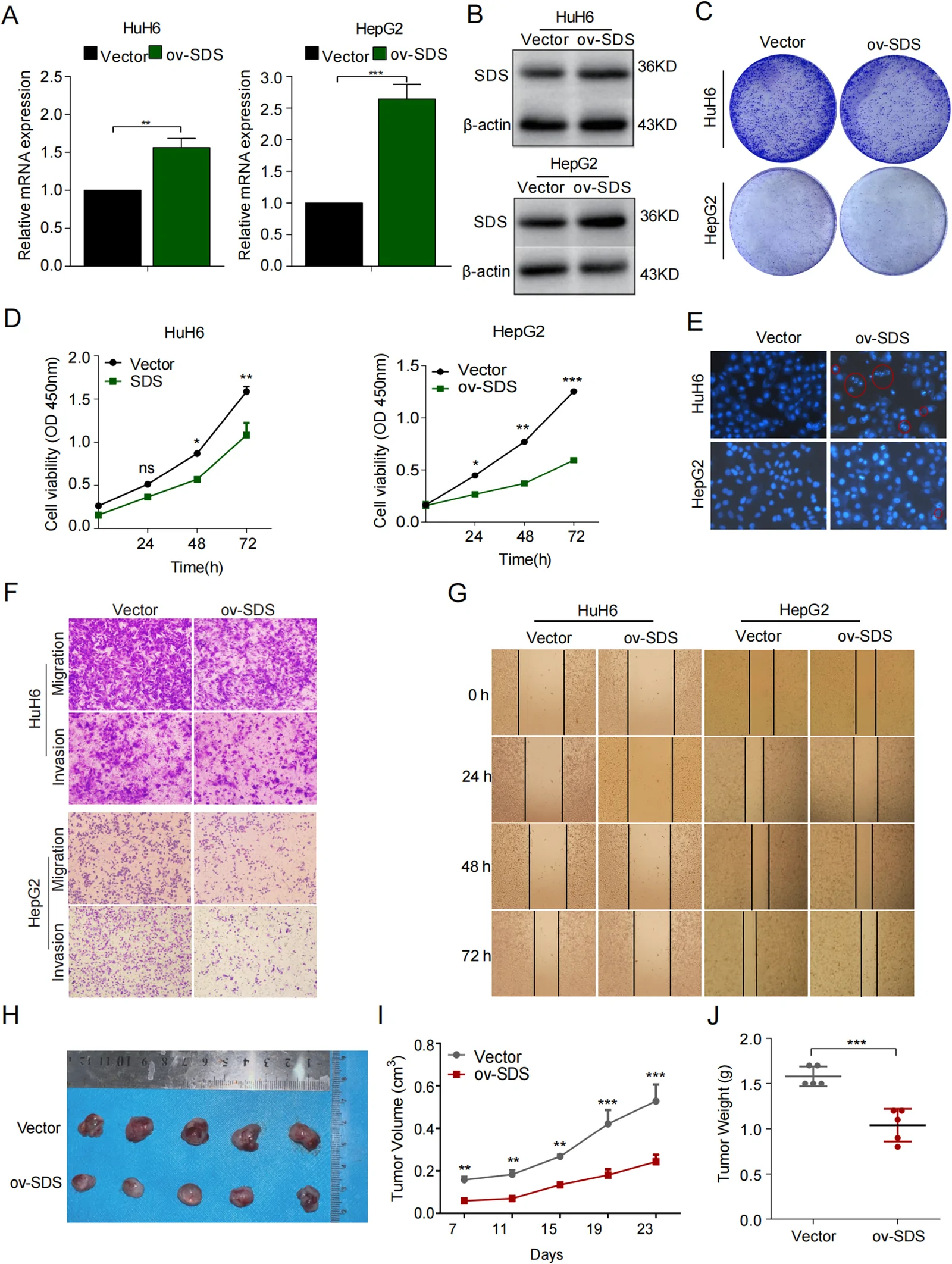
SDS overexpression inhibition the growth and metastasis of hepatoblastoma. **(A)** qRT-PCR detection of overexpression efficiency of SDS. **(B)** Western Blot detection of overexpression efficiency of SDS. **(C)** Colony formation assays in hepatoblastoma cells. **(D)** CCK-8 assay cell viability in hepatoblastoma cells. **(E)** Hoechst33324 staining detects the effect of upregulation of SDS on cell apoptosis. **(F)** Transwell assays the migration and invasion in hepatoblastoma cells. **(G)** Detection of cell migration in hepatoblastoma cells of SDS overexpression treatment using wound healing experiment. **(H)** Representative image of tumors formed in nude mice. **(I)** The subcutaneous tumor volume growth curves of different groups. **(J)** Subcutaneous weight of tumor in nude mice. Data are the mean ± SD. from three independent experiments. ^*^P < 0.05, ^**^P < 0.01, ^***^P < 0.001; ns, not significant.

## 4 Discussion

HB is the most common primary liver malignancy in children. The prognosis of high-risk HB patients remains unsatisfactory, largely due to the absence of robust molecular biomarkers for precise risk stratification and individualized therapy. To address this issue, we integrated RF, LASSO and SVM-RFE to screen four central genes, CLEC1B, ITGA6, SDS, and TTC36, as key diagnostic biomarkers for HB. Based on these four genes, consensus clustering analysis was further performed on 146 HB tissue samples, classifying them into two molecular subtypes with distinct immune activation profiles. Functional experiments demonstrated that SDS overexpression significantly suppressed HB cell proliferation, migration, invasion, and colony formation while promoting apoptosis, suggesting that SDS acts as a novel tumor suppressor in HB.

A notable methodological strength of this study is the integration of multiple machine learning algorithms for biomarker discovery. Machine learning has recently found wide application in the identification of diagnostic and prognostic markers in various malignancies. LASSO regression is extensively used for its ability to perform feature selection in high-dimensional data by shrinking coefficients of irrelevant predictors to zero. For instance, LASSO regression with 10-fold cross-validation was applied to select 22 imaging features associated with bladder cancer, demonstrating the practical value of machine learning for histopathological image analysis in a clinical setting (21). RF is an ensemble method that aggregates predictions from multiple decision trees and robustly assesses feature importance even in noisy datasets. It has been successfully used to construct a radiomics-based model reflecting the tumor microenvironment phenotype, which predicts clinical response to anti-PD-1/PD-L1 immunotherapy in breast cancer (22). SVM-RFE selects the optimal variable subset by iteratively removing the least important features; it has been applied to radiomics-based molecular subtyping of lung adenocarcinoma, revealing associations between distinct radiomics clusters and genomic, transcriptomic, and immune microenvironment features (23). Notably, integrated strategies combining multiple machine learning algorithms have demonstrated significantly improved predictive performance compared to single-algorithm approaches or traditional models based solely on clinical data (20). Consistent with these findings, our study combined random forest, LASSO, and SVM-RFE for hub gene screening. Overlapping the results from the three algorithms revealed four hub genes with strong diagnostic performance, further validating the unique advantage of multi-algorithm integration in biomarker discovery.

CLEC1B encodes a protein involved in platelet activation, tumor cell metastasis, and blood/lymphatic vessel separation (24–26). This gene is found not just in platelets but also in various immune cells, including myeloid cells, dendritic cells, macrophages, and natural killer cells (24). There has been growing interest in the role of CLEC1B in immune and inflammatory responses over recent years. Hu et al. found that CLEC1B and PD-L1 expression jointly hold prognostic significance for response to ICB therapy (27). Moreover, CLEC-2 (encoded by CLEC1B) also plays a critical role in supporting lymphocyte proliferation and preserving lymph node homeostasis, and the migration of dendritic cells to lymph nodes depends on dendritic cell-specific expression of CLEC-2 (28–30). ITGA6 (integrin α6) is widely involved in multiple biological processes including cell adhesion, stemness maintenance, migration, invasion, immune evasion, angiogenesis, and drug resistance. It has been recognized as a potential diagnostic marker and therapeutic target in various tumors (31–34). Targeted inhibition of ITGA6 regulates the production and release of insulin-like growth factors (IGFs), thereby preventing the dissemination of platinum-resistant epithelial ovarian cancer and enhancing its sensitivity to platinum-based drugs (35). Furthermore, inhibition of ITGA6 not only overcomes radiation resistance in mesenchymal glioblastoma (GBM)-like stem cells but also reduces the proliferation and stemness of proneural GBM-like stem cells, suggesting that ITGA6 is a potential therapeutic target for improving GBM patient prognosis (36). In hepatocellular carcinoma (HCC), ITGA6 exerts a pro-malignant effect through the formation of the integrin α6β4 complex (37). TTC36 (tetratricopeptide repeat domain 36) functions as a molecular chaperone of heat shock protein 70. Several recent studies have revealed a dual role of TTC36 in HCC. On one hand, TTC36 upregulates SPRED1 by stabilizing YBX3, thereby inhibiting Ras/MAPK-driven HCC cell proliferation (38). On the other hand, TTC36 promotes SET-mediated inhibition of PP2A activity, thereby enhancing c-Myc stability via the AKT/GSK3β axis, which ultimately suppresses HCC cell proliferation and increases sensitivity to sorafenib (39).

SDS (serine dehydratase) catalyzes the deamination of L-serine to pyruvate and ammonia, serving as a key node in the glycine, serine, and threonine metabolic network. In mammals, the liver is the central organ for SDS-regulated serine metabolism; this enzyme participates in gluconeogenesis and coordinates systemic amino acid and sphingolipid homeostasis (40). In serine-auxotrophic tumors (e.g., luminal/estrogen receptor-positive breast cancer), engineered SDS exhibits tumor-suppressive effects both *in vitro* and *in vivo (*41). Furthermore, limiting serine supply in colorectal cancer cells has been shown to cause mitochondrial dysfunction and cytoplasmic accumulation of mitochondrial DNA, which then activates the cGAS-STING pathway, promotes type I interferon release, and increases tumor immunogenicity as well as sensitivity to immune checkpoint blockade (42). Consistent with these observations, when we analyzed the association of SDS expression with the HB immune microenvironment, we found that SDS levels correlated positively with several immune-infiltrating cell types, particularly dendritic cells, Th1 cells, and macrophages. This pattern resonates with emerging evidence that metabolic regulators can shape the tumor immune landscape through chemokine-mediated immune cell trafficking. For example, Song et al. recently demonstrated that anlotinib suppresses transferrin receptor (TFRC) expression via the VEGFR2/AKT/HIF-1α axis in HCC, leading to CXCL14 upregulation and subsequent CD8^+^ T-cell infiltration, thereby potentiating anti-PD-1 immunotherapy (43). Although our results indicate that SDS not only regulates the biological functions of HB but also associates with tumor-infiltrating immune cells, the mechanisms by which SDS triggers anti-tumor immune cell infiltration remain unresolved and warrant further investigation.

From the perspective of clinical translation and pathological practice, the findings of this study hold multi-level potential value. At the diagnostic level, the four-gene panel (CLEC1B, ITGA6, SDS, and TTC36) can serve as a molecular adjunct to histopathological examination, thereby improving the diagnostic accuracy of hepatoblastoma. In terms of subtyping and prognostic assessment, the two molecular subtypes (Clust1 and Clust2) identified by consensus clustering exhibit markedly distinct immune infiltration profiles and pathway activation patterns, enabling molecular-based prognostic stratification of patients. Notably, SDS expression levels correlate positively with immune scores and the degree of infiltration of multiple immune cell types; its low expression suggests enhanced tumor proliferative activity and an unfavorable prognostic tendency, rendering it a useful auxiliary indicator for prognosis. Regarding therapeutic decision-making, patients with high SDS expression are predominantly enriched in immune-related pathways and may therefore be more responsive to immune checkpoint inhibitor therapy, whereas those with low SDS expression show enrichment in cell cycle and proliferation-related pathways, potentially making them better candidates for anti-proliferative chemotherapeutic agents. Furthermore, as a key enzyme in the serine metabolic pathway, SDS exerts tumor-suppressive functions, suggesting that targeting SDS-mediated metabolic reprogramming may represent a novel therapeutic strategy. Collectively, our model provides a tool that integrates molecular diagnosis, prognostic assessment, and therapeutic guidance, while also offering a theoretical foundation and potential intervention targets for precision medicine and individualized treatment decision-making in hepatoblastoma.

Several limitations of this study should be acknowledged when interpreting our findings. First, it should be acknowledged that all data utilized in this study were obtained from publicly available GEO databases. While these datasets have provided us with a relatively large sample size, they unfortunately do not contain detailed clinicopathological information, such as histological subtypes, tumor stage, or prognostic outcomes. We fully recognize this as an important limitation of the current study. Second, the precise molecular mechanisms through which SDS suppresses hepatoblastoma progression and modulates the immune microenvironment remain incompletely elucidated. While our functional experiments have confirmed the tumor-suppressive role of SDS, further mechanistic investigations are required to decipher how SDS influences the biological behavior of HB cells and interferes with immune cell functions. This line of inquiry may offer a novel theoretical foundation for immunotherapy in hepatoblastoma, and it constitutes a primary focus of our ongoing research. Looking forward, we plan to conduct prospective cohort studies incorporating clinical tissue specimens, longitudinal follow-up data, and histopathological images from representative cases to validate and extend our findings.

## 5 Conclusion

By integrating bioinformatics and machine learning approaches, this study identified four hub genes (CLEC1B, ITGA6, SDS, and TTC36) with high diagnostic value for hepatoblastoma. Among them, SDS is not only strongly correlated with immune infiltration but also suppresses the malignant phenotype of HB cells, making it a promising biomarker and functional tumor suppressor. These findings provide new insights into the molecular subtyping of HB and lay a theoretical foundation for developing novel diagnostic and therapeutic strategies targeting serine metabolism and immune regulation.

## Data Availability

The original contributions presented in the study are included in the article/**Supplementary Material**. Further inquiries can be directed to the corresponding author.

## Supplementary material

SUPPLEMENTARY FIGURE 1 **(A)** Distribution of data before and after batch effect removal. **(B)** Umap distribution before and after batch effect removal.

SUPPLEMENTARY FIGURE 2 **(A)** ROC curves for evaluating the diagnostic performance of the alpha-fetoprotein. **(B-E)** ROC curves for evaluating the diagnostic performance of the CLEC, ITGA6, SDS, TTC36.

SUPPLEMENTARY FIGURE 3 **(A)** Enrichment of apoptotic pathways in HALLMARK and KEGG. **(B)** Enrichment of EMT and cell adhesion pathways in HALLMARK and KEGG.

SUPPLEMENTARY FIGURE 4 **(A)** Enrichment of apoptotic pathways in HALLMARK and KEGG. **(B)** Enrichment of EMT and cell adhesion pathways in HALLMARK and KEGG.
